# Dichlorido(6,6′-dimethyl-2,2′-bipyridine-κ^2^ *N*,*N*′)cadmium(II)

**DOI:** 10.1107/S1600536810029399

**Published:** 2010-07-31

**Authors:** Robabeh Alizadeh, Parisa Mohammadi Eshlaghi, Vahid Amani

**Affiliations:** a School of Chemistry, Damghan University, Damghan, Iran; b Islamic Azad University, Shahr-e-Rey Branch, Tehran, Iran

## Abstract

In the title compound, [CdCl_2_(C_12_H_12_N_2_)], the Cd^II^ atom is four-coordinated in a distorted tetra­hedral geometry by two N atoms from a 6,6′-dimethyl-2,2′-bipyridine ligand and two terminal Cl atoms. Inter­molecular C—H⋯Cl hydrogen bonds and π–π stacking inter­actions between the pyridyl rings [centroid–centroid distance = 3.7337 (18) Å] are present in the crystal structure.

## Related literature

For related structures, see: Alizadeh, Kalateh, Ebadi *et al.* (2009 ▶); Alizadeh, Kalateh, Khoshtarkib *et al.* (2009 ▶); Alizadeh, Khoshtarkib *et al.* (2009 ▶); Itoh *et al.* (2005 ▶); Kou *et al.* (2008 ▶); Onggo *et al.* (2005 ▶).
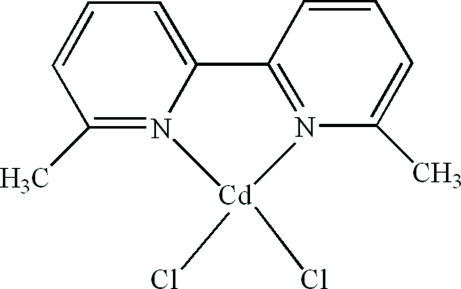


## Experimental

### 

#### Crystal data

[CdCl_2_(C_12_H_12_N_2_)]
*M* *_r_* = 367.55
Monoclinic, 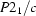

*a* = 7.6715 (9) Å
*b* = 10.0970 (16) Å
*c* = 17.902 (2) Å
β = 97.474 (9)°
*V* = 1374.9 (3) Å^3^
*Z* = 4
Mo *K*α radiation
μ = 1.96 mm^−1^
*T* = 298 K
0.50 × 0.25 × 0.17 mm

#### Data collection

Bruker SMART APEX CCD diffractometer
Absorption correction: multi-scan (*SADABS*; Sheldrick, 1996 ▶) *T*_min_ = 0.569, *T*_max_ = 0.723
9684 measured reflections
3656 independent reflections
3162 reflections with *I* > 2σ(*I*)
*R*_int_ = 0.045

#### Refinement

*R*[*F* ^2^ > 2σ(*F* ^2^)] = 0.033
*wR*(*F* ^2^) = 0.086
*S* = 1.08
3656 reflections
154 parameters
H-atom parameters constrained
Δρ_max_ = 0.57 e Å^−3^
Δρ_min_ = −0.63 e Å^−3^

### 

Data collection: *SMART* (Bruker, 2007 ▶); cell refinement: *SAINT* (Bruker, 2007 ▶); data reduction: *SAINT*; program(s) used to solve structure: *SHELXTL* (Sheldrick, 2008 ▶); program(s) used to refine structure: *SHELXTL*; molecular graphics: *ORTEP-3* (Farrugia, 1997 ▶); software used to prepare material for publication: *WinGX* (Farrugia, 1999 ▶).

## Supplementary Material

Crystal structure: contains datablocks I. DOI: 10.1107/S1600536810029399/hy2335sup1.cif

Structure factors: contains datablocks I. DOI: 10.1107/S1600536810029399/hy2335Isup2.hkl

Additional supplementary materials:  crystallographic information; 3D view; checkCIF report

## Figures and Tables

**Table 1:** Selected bond lengths (Å)

Cd1—N1	2.268 (2)
Cd1—N2	2.2752 (19)
Cd1—Cl1	2.3919 (9)
Cd1—Cl2	2.3885 (8)

**Table 2:** Hydrogen-bond geometry (Å, °)

*D*—H⋯*A*	*D*—H	H⋯*A*	*D*⋯*A*	*D*—H⋯*A*
C1—H1*C*⋯Cl1^i^	0.96	2.76	3.711 (4)	169
C5—H5⋯Cl1^ii^	0.93	2.79	3.551 (3)	140

Symmetry codes: (i) 
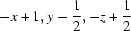
; (ii) 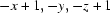.

**Fig. 1.**
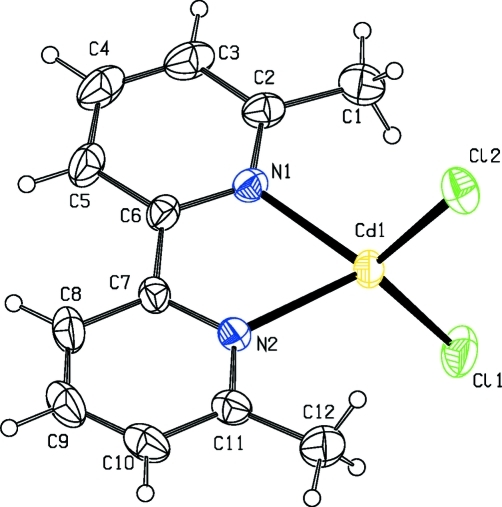
The molecular structure of the title compound. Displacement ellipsoids are drawn at the 30% probability level.

**Fig. 2.**
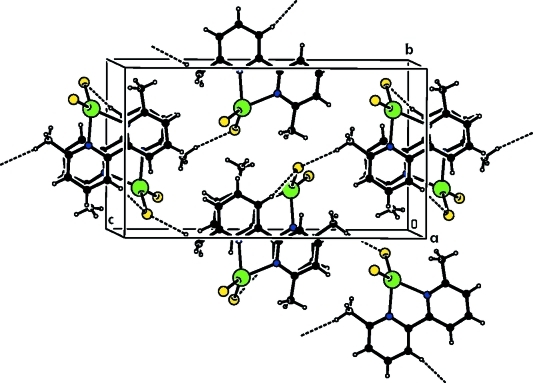
Crystal packing diagram of the title compound. Dashed lines denote hydrogen bonds.

## supplementary crystallographic information

### Comment

6,6'-Dimethyl-2,2'-bipyridine (6,6'-dmbipy) is a good bidentate ligand, and numerous complexes with 6,6'-dmbipy have been prepared, such as those of zinc (Alizadeh, Kalateh, Ebadi *et al.*, 2009; Alizadeh, Kalateh, Khoshtarkib, *et al.*, 2009; Alizadeh, Khoshtarkib *et al.*, 2009), copper (Itoh *et al.*, 2005), nickel (Kou *et al.*, 2008) and ruthenium (Onggo *et al.*, 2005). We report herein the synthesis and crystal structure of the title compound.

In the title compound (Fig. 1), the Cd ^II^ atom is four-coordinated in a distorted tetrahedral geometry by two N atoms from a 6,6'-dmbipy ligand and two terminal Cl atoms. The Cd—N and Cd—Cl bond lengths and angles are normal (Table 1). In the crystal structure, intermolecular C—H···Cl hydrogen bonds (Table 2) and π–π contacts (Fig. 2) between the pyridyl rings, *Cg*1···*Cg*2^i^ [symmetry code: (i) 1-x, -y, 1-z. *Cg*1 and *Cg*2 are centroids of the N1, C2—C6 ring and the N2, C7—C11 ring], stabilize the structure, with a centroid–centroid distance of 3.7337 (18) Å.

### Experimental

A solution of 6,6'-dimethyl-2,2'-bipyridine (0.25 g, 1.33 mmol) in methanol (10 ml) was added to a solution of CdCl_2_.H_2_O (0.27 g, 1.33 mmol) in methanol (5 ml) at room temperature. Crystals suitable for X-ray diffraction experiment were obtained by methanol diffusion into a colorless solution of the title compound in DMSO after one week (yield: 0.35 g, 71.6%).

### Refinement

H atoms were positioned geometrically and refined as riding atoms, with C—H = 0.93 (aromatic) and 0.96 (methyl) Å and with *U*_iso_(H) = 1.2*U*_eq_(C).

### Crystal data

**Table:**

[CdCl_2_(C_12_H_12_N_2_)]	*F*(000) = 720
*M**_r_* = 367.55	*D*_x_ = 1.776 Mg m^−^^3^
Monoclinic, *P*2_1_/*c*	Mo *K*α radiation, λ = 0.71073 Å
Hall symbol: -P 2ybc	Cell parameters from 899 reflections
*a* = 7.6715 (9) Å	θ = 2.3–29.2°
*b* = 10.0970 (16) Å	µ = 1.96 mm^−^^1^
*c* = 17.902 (2) Å	*T* = 298 K
β = 97.474 (9)°	Block, colorless
*V* = 1374.9 (3) Å^3^	0.50 × 0.25 × 0.17 mm
*Z* = 4	

### Data collection

**Table:**

Bruker APEX CCD diffractometer	3656 independent reflections
Radiation source: fine-focus sealed tube	3162 reflections with *I* > 2σ(*I*)
graphite	*R*_int_ = 0.045
φ and ω scans	θ_max_ = 29.2°, θ_min_ = 2.3°
Absorption correction: multi-scan (*SADABS*; Sheldrick, 1996)	*h* = −10→10
*T*_min_ = 0.569, *T*_max_ = 0.723	*k* = −11→13
9684 measured reflections	*l* = −24→24

### Refinement

**Table:**

Refinement on *F*^2^	Primary atom site location: structure-invariant direct methods
Least-squares matrix: full	Secondary atom site location: difference Fourier map
*R*[*F*^2^ > 2σ(*F*^2^)] = 0.033	Hydrogen site location: inferred from neighbouring sites
*wR*(*F*^2^) = 0.086	H-atom parameters constrained
*S* = 1.08	*w* = 1/[σ^2^(*F*_o_^2^) + (0.0364*P*)^2^ + 0.7364*P*] where *P* = (*F*_o_^2^ + 2*F*_c_^2^)/3
3656 reflections	(Δ/σ)_max_ = 0.001
154 parameters	Δρ_max_ = 0.57 e Å^−^^3^
0 restraints	Δρ_min_ = −0.63 e Å^−^^3^

### Fractional atomic coordinates and isotropic or equivalent isotropic displacement parameters (Å^2^)

**Table:**

	*x*	*y*	*z*	*U*_iso_*/*U*_eq_	
C1	0.3527 (5)	0.0531 (4)	0.26805 (18)	0.0737 (9)	
H1A	0.4386	0.1215	0.2805	0.088*	
H1B	0.2400	0.0927	0.2526	0.088*	
H1C	0.3861	0.0002	0.2277	0.088*	
C2	0.3427 (4)	−0.0323 (3)	0.33533 (16)	0.0526 (6)	
C3	0.3765 (4)	−0.1659 (3)	0.3346 (2)	0.0690 (9)	
H3	0.4058	−0.2060	0.2912	0.083*	
C4	0.3668 (5)	−0.2393 (3)	0.3977 (3)	0.0754 (11)	
H4	0.3912	−0.3296	0.3978	0.090*	
C5	0.3202 (4)	−0.1785 (3)	0.4621 (2)	0.0634 (8)	
H5	0.3131	−0.2276	0.5055	0.076*	
C6	0.2846 (3)	−0.0442 (2)	0.46048 (15)	0.0451 (5)	
C7	0.2319 (3)	0.0282 (3)	0.52617 (13)	0.0435 (5)	
C8	0.2145 (4)	−0.0343 (3)	0.59409 (17)	0.0613 (8)	
H8	0.2401	−0.1239	0.6005	0.074*	
C9	0.1589 (5)	0.0381 (4)	0.65159 (17)	0.0698 (9)	
H9	0.1458	−0.0026	0.6971	0.084*	
C10	0.1233 (4)	0.1690 (4)	0.64177 (16)	0.0638 (8)	
H10	0.0852	0.2183	0.6804	0.077*	
C11	0.1442 (4)	0.2293 (3)	0.57342 (15)	0.0512 (6)	
C12	0.1080 (6)	0.3718 (3)	0.5588 (2)	0.0744 (9)	
H12A	0.0164	0.3811	0.5172	0.089*	
H12B	0.2127	0.4146	0.5469	0.089*	
H12C	0.0715	0.4120	0.6028	0.089*	
N1	0.2975 (3)	0.0269 (2)	0.39745 (11)	0.0432 (4)	
N2	0.1971 (3)	0.1583 (2)	0.51688 (10)	0.0416 (4)	
Cd1	0.23178 (3)	0.245465 (17)	0.402558 (10)	0.04588 (8)	
Cl1	0.47640 (12)	0.38850 (9)	0.39295 (5)	0.0758 (2)	
Cl2	−0.03250 (11)	0.31365 (9)	0.32689 (4)	0.0646 (2)	

### Atomic displacement parameters (Å^2^)

**Table:**

	*U*^11^	*U*^22^	*U*^33^	*U*^12^	*U*^13^	*U*^23^
C1	0.098 (3)	0.074 (2)	0.0528 (16)	0.0060 (19)	0.0223 (16)	−0.0106 (15)
C2	0.0483 (14)	0.0502 (15)	0.0586 (15)	0.0060 (11)	0.0044 (11)	−0.0126 (12)
C3	0.0651 (18)	0.0542 (17)	0.086 (2)	0.0132 (14)	0.0011 (16)	−0.0255 (17)
C4	0.068 (2)	0.0414 (16)	0.112 (3)	0.0141 (13)	−0.006 (2)	−0.0137 (16)
C5	0.0637 (17)	0.0381 (14)	0.084 (2)	0.0048 (12)	−0.0088 (15)	0.0090 (13)
C6	0.0398 (12)	0.0360 (11)	0.0563 (13)	−0.0014 (9)	−0.0060 (10)	0.0041 (10)
C7	0.0396 (11)	0.0452 (13)	0.0438 (12)	−0.0055 (9)	−0.0017 (9)	0.0094 (10)
C8	0.0597 (16)	0.0636 (18)	0.0590 (16)	−0.0095 (14)	0.0019 (13)	0.0260 (14)
C9	0.0691 (19)	0.096 (3)	0.0444 (14)	−0.0182 (18)	0.0075 (13)	0.0219 (16)
C10	0.0610 (17)	0.092 (3)	0.0399 (13)	−0.0105 (16)	0.0103 (11)	−0.0033 (14)
C11	0.0536 (14)	0.0589 (16)	0.0417 (12)	−0.0037 (12)	0.0078 (10)	−0.0053 (11)
C12	0.106 (3)	0.0579 (19)	0.0631 (18)	0.0082 (18)	0.0232 (18)	−0.0126 (15)
N1	0.0445 (10)	0.0377 (10)	0.0469 (10)	0.0011 (8)	0.0041 (8)	−0.0034 (8)
N2	0.0452 (10)	0.0430 (10)	0.0363 (9)	−0.0023 (8)	0.0040 (7)	0.0022 (8)
Cd1	0.05923 (13)	0.03760 (12)	0.04198 (11)	0.00404 (7)	0.01099 (8)	0.00679 (6)
Cl1	0.0785 (5)	0.0607 (5)	0.0887 (6)	−0.0146 (4)	0.0127 (4)	0.0244 (4)
Cl2	0.0668 (4)	0.0701 (5)	0.0562 (4)	0.0142 (4)	0.0048 (3)	0.0153 (3)

### Geometric parameters (Å, °)

**Table:**

C1—C2	1.491 (5)	C8—C9	1.374 (5)
C1—H1A	0.9600	C8—H8	0.9300
C1—H1B	0.9600	C9—C10	1.357 (6)
C1—H1C	0.9600	C9—H9	0.9300
C2—N1	1.347 (3)	C10—C11	1.394 (4)
C2—C3	1.374 (4)	C10—H10	0.9300
C3—C4	1.361 (6)	C11—N2	1.346 (3)
C3—H3	0.9300	C11—C12	1.482 (4)
C4—C5	1.394 (6)	C12—H12A	0.9600
C4—H4	0.9300	C12—H12B	0.9600
C5—C6	1.383 (4)	C12—H12C	0.9600
C5—H5	0.9300	Cd1—N1	2.268 (2)
C6—N1	1.352 (3)	Cd1—N2	2.2752 (19)
C6—C7	1.485 (4)	Cd1—Cl1	2.3919 (9)
C7—N2	1.345 (3)	Cd1—Cl2	2.3885 (8)
C7—C8	1.392 (3)		
			
C2—C1—H1A	109.5	C10—C9—C8	119.9 (3)
C2—C1—H1B	109.5	C10—C9—H9	120.1
H1A—C1—H1B	109.5	C8—C9—H9	120.1
C2—C1—H1C	109.5	C9—C10—C11	119.7 (3)
H1A—C1—H1C	109.5	C9—C10—H10	120.2
H1B—C1—H1C	109.5	C11—C10—H10	120.2
N1—C2—C3	120.8 (3)	N2—C11—C10	120.5 (3)
N1—C2—C1	117.1 (3)	N2—C11—C12	116.9 (3)
C3—C2—C1	122.0 (3)	C10—C11—C12	122.6 (3)
C4—C3—C2	119.8 (3)	C11—C12—H12A	109.5
C4—C3—H3	120.1	C11—C12—H12B	109.5
C2—C3—H3	120.1	H12A—C12—H12B	109.5
C3—C4—C5	119.6 (3)	C11—C12—H12C	109.5
C3—C4—H4	120.2	H12A—C12—H12C	109.5
C5—C4—H4	120.2	H12B—C12—H12C	109.5
C6—C5—C4	119.1 (3)	C2—N1—C6	120.6 (2)
C6—C5—H5	120.5	C2—N1—Cd1	123.18 (18)
C4—C5—H5	120.5	C6—N1—Cd1	116.24 (16)
N1—C6—C5	120.1 (3)	C7—N2—C11	120.1 (2)
N1—C6—C7	117.2 (2)	C7—N2—Cd1	116.38 (16)
C5—C6—C7	122.7 (3)	C11—N2—Cd1	123.52 (18)
N2—C7—C8	120.7 (3)	N1—Cd1—N2	73.28 (8)
N2—C7—C6	116.9 (2)	N1—Cd1—Cl2	115.75 (6)
C8—C7—C6	122.4 (3)	N2—Cd1—Cl2	115.59 (6)
C9—C8—C7	119.2 (3)	N1—Cd1—Cl1	113.82 (6)
C9—C8—H8	120.4	N2—Cd1—Cl1	118.88 (6)
C7—C8—H8	120.4	Cl2—Cd1—Cl1	113.65 (3)
			
N1—C2—C3—C4	−1.0 (5)	C5—C6—N1—Cd1	179.2 (2)
C1—C2—C3—C4	179.5 (3)	C7—C6—N1—Cd1	−1.0 (3)
C2—C3—C4—C5	1.0 (5)	C8—C7—N2—C11	−0.3 (4)
C3—C4—C5—C6	0.0 (5)	C6—C7—N2—C11	178.2 (2)
C4—C5—C6—N1	−1.0 (4)	C8—C7—N2—Cd1	−179.97 (19)
C4—C5—C6—C7	179.2 (3)	C6—C7—N2—Cd1	−1.4 (3)
N1—C6—C7—N2	1.6 (3)	C10—C11—N2—C7	−0.5 (4)
C5—C6—C7—N2	−178.6 (2)	C12—C11—N2—C7	−179.8 (3)
N1—C6—C7—C8	−179.9 (2)	C10—C11—N2—Cd1	179.1 (2)
C5—C6—C7—C8	−0.1 (4)	C12—C11—N2—Cd1	−0.2 (4)
N2—C7—C8—C9	0.9 (4)	C2—N1—Cd1—N2	178.4 (2)
C6—C7—C8—C9	−177.6 (3)	C6—N1—Cd1—N2	0.18 (16)
C7—C8—C9—C10	−0.5 (5)	C2—N1—Cd1—Cl2	67.6 (2)
C8—C9—C10—C11	−0.3 (5)	C6—N1—Cd1—Cl2	−110.67 (16)
C9—C10—C11—N2	0.8 (5)	C2—N1—Cd1—Cl1	−66.8 (2)
C9—C10—C11—C12	−179.9 (3)	C6—N1—Cd1—Cl1	114.92 (16)
C3—C2—N1—C6	0.1 (4)	C7—N2—Cd1—N1	0.69 (16)
C1—C2—N1—C6	179.5 (3)	C11—N2—Cd1—N1	−179.0 (2)
C3—C2—N1—Cd1	−178.1 (2)	C7—N2—Cd1—Cl2	111.73 (16)
C1—C2—N1—Cd1	1.4 (4)	C11—N2—Cd1—Cl2	−67.9 (2)
C5—C6—N1—C2	0.9 (4)	C7—N2—Cd1—Cl1	−107.72 (16)
C7—C6—N1—C2	−179.3 (2)	C11—N2—Cd1—Cl1	72.6 (2)

### Hydrogen-bond geometry (Å, °)

**Table:**

*D*—H···*A*	*D*—H	H···*A*	*D*···*A*	*D*—H···*A*
C1—H1C···Cl1^i^	0.96	2.76	3.711 (4)	169
C5—H5···Cl1^ii^	0.93	2.79	3.551 (3)	140

Symmetry codes: (i) −*x*+1, *y*−1/2, −*z*+1/2; (ii) −*x*+1, −*y*, −*z*+1.
